# Identification of human MHC-I HPV18 E6/E7-specific CD8 + T cell epitopes and generation of an HPV18 E6/E7-expressing adenosquamous carcinoma in HLA-A2 transgenic mice

**DOI:** 10.1186/s12929-022-00864-5

**Published:** 2022-10-12

**Authors:** Shiwen Peng, Deyin Xing, Louise Ferrall, Ya-Chea Tsai, Chien-Fu Hung, T.-C. Wu

**Affiliations:** 1 Department of Pathology, The Johns Hopkins University, Baltimore, MD USA; 2 Department of Oncology, The Johns Hopkins University, Baltimore, MD USA; 3 Department of Obstetrics and Gynecology, The Johns Hopkins University, Baltimore, MD USA; 4 Department of Molecular Microbiology and Immunology, The Johns Hopkins University, Baltimore, MD USA; 5 The Johns Hopkins Medical Institutions, CRB II Room 307, 1550 Orleans St., Baltimore, MD 21231 USA; 6 The Johns Hopkins Medical Institutions, CRB II Room 309, 1550 Orleans St., Baltimore, MD 21231 USA

**Keywords:** HPV18, HLA-A2, Adenosquamous cell carcinoma, Mouse model, Epitope, E6, E7

## Abstract

**Background:**

Human Papillomavirus type 18 (HPV18) is a high-risk HPV that is commonly associated with cervical cancer. HPV18 oncogenes E6 and E7 are associated with the malignant transformation of cells, thus the identification of human leukocyte antigen (HLA)-restricted E6/E7 peptide-specific CD8 + T cell epitopes and the creation of a HPV18 E6/E7 expressing cervicovaginal tumor in HLA-A2 transgenic mice will be significant for vaccine development.

**Methods:**

In the below study, we characterized various human HLA class I-restricted HPV18 E6 and E7-specific CD8 + T cells mediated immune responses in HLA class I transgenic mice using DNA vaccines encoding HPV18E6 and HPV18E7. We then confirmed HLA-restricted E6/E7 specific CD8 + T cell epitopes using splenocytes from vaccinated mice stimulated with HPV18E6/E7 peptides. Furthermore, we used oncogenic DNA plasmids encoding HPV18E7E6(delD70), luciferase, cMyc, and AKT to create a spontaneous cervicovaginal carcinoma model in HLA-A2 transgenic mice.

**Results:**

Therapeutic HPV18 E7 DNA vaccination did not elicit any significant CD8 + T cell response in HLA-A1, HLA-24, HLA-B7, HLA-B44 transgenic or wild type C57BL/6 mice, but it did generate a strong HLA-A2 and HLA-A11 restricted HPV18E7-specific CD8 + T cell immune response. We found that a single deletion of aspartic acid (D) at location 70 in HPV18E6 DNA abolishes the presentation of HPV18 E6 peptide (aa67-75) by murine MHC class I. We found that the DNA vaccine with this mutant HPV18 E6 generated E6-specific CD8 + T cells in HLA-A2. HLA-A11, HLA-A24 and HLA-b40 transgenic mice. Of note, HLA-A2 restricted, HPV18 E7 peptide (aa7-15)- and HPV18 E6 peptide (aa97-105)-specific epitopes are endogenously processed by HPV18 positive Hela-AAD (HLA-A^*^0201/D^d^) cells. Finally, we found that injection of DNA plasmids encoding HPV18E7E6(delD70), AKT, cMyc, and SB100 can result in the development of adenosquamous carcinoma in the cervicovaginal tract of HLA-A2 transgenic mice.

**Conclusions:**

We characterized various human HLA class I-restricted HPV18 E6/E7 peptide specific CD8 + T cell epitopes in human HLA class I transgenic mice. We demonstrated that HPV18 positive Hela cells expressing chimeric HLA-A2 (AAD) do present both HLA-A2-restricted HPV18 E7 (aa7-15)- and HPV18 E6 (aa97-105)-specific CD8 + T cell epitopes. A mutant HPV18E6 that had a single deletion at location 70 obliterates the E6 presentation by murine MHC class I and remains oncogenic. The identification of these human MHC restricted HPV antigen specific epitopes as well as the HPV18E6/E7 expressing adenosquamous cell carcinoma model may have significant future translational potential.

**Supplementary Information:**

The online version contains supplementary material available at 10.1186/s12929-022-00864-5.

## Background

Human Papillomavirus (HPV) is a sexually transmitted virus that is a common etiological factor in several human cancers, especially high-risk HPV types (hrHPV) HPV16 and HPV18. HPV-associated malignancies include cervical, anal, penile, vulvar, vaginal, and oropharyngeal or head and neck cancers [1]. Although there are commercially available preventative vaccines [2–4], these prophylactic vaccines are unable to clear existing infection. Of existing HPV infections, a percentage will progress to lesions and potentially to malignant cancers. Although some HPV-associated cancers can be treated in their early stages, patients with metastatic or advanced HPV-associated cancers therapeutic options have limited success and can come with significant morbidity. Therapeutic HPV vaccines represent a promising alternative treatment strategy to clear high-risk HPV infections and associated malignancies, because they can target HPV-associated tissues and infections that are already established.

HPV has two dominant oncogenic genes: E6 and E7. E6 and E7 are thus ideal immunotherapeutic targets for the development of therapeutic HPV vaccines for several reasons: (1) E6/E7 oncogenic proteins are typically required for the initiation and maintenance of HPV associated malignancies, rendering them essential for the cancer growth and preventing immune escape [5]; (2) E6/E7 are consistently expressed in HPV-associated malignancies and HPV-infected cells, but not in healthy cells, providing tumor specificity [6, 7]; and (3) E6/E7 oncogenic proteins are foreign viral antigens and are not subject to central tolerance by human immune systems. Taken together, both E6 and E7 represent ideal antigens for the development of therapeutic HPV vaccines. We have developed several therapeutic HPV vaccines, including a DNA vaccine encoding calreticulin (CRT) linked to HPV-16 E7 antigen (CRT/E7) [8]. In addition, we have developed a DNA vaccine encoding mycobacteria heat shock protein 70 linked to the E6 and E7 proteins from HPV16 and HPV18 called pBI-11 [9]. Furthermore, we have used therapeutic HPV vaccinia vaccine (TA-HPV) to boost the immune responses generated by therapeutic HPV DNA vaccine in a DNA prime vaccinia boost regimen [9–11].

The identification of human major histocompatibility (MHC) class I restricted HPV E6/E7 antigen-specific CD8 + T cell epitopes will be important for therapeutic HPV vaccine development. The term “epitope” represents the antigenic peptide that can be presented by particular MHC class I molecule on antigen presenting cells to CD8 + T cells. The characterization of these CD8 + T cell epitopes will additionally be helpful for the development of quantitative CD8 + T cell mediated immunological assays to characterize HPV antigen-specific CD8 + T cell-mediated immune responses, thereby facilitating the development of vaccines and/or immunotherapeutic strategies against HPV-associated lesions. Peptide vaccines specifically require known CD8 + T cell epitopes specific for the patient’s MHC class I type. Because different human MHC class I alleles can potentially present different regions of the E6/E7 proteins to CD8 + T cells, it is important to identify different epitopes of E6/E7 proteins that are presented by commonly expressed human MHC class I molecules. It has been shown that human leukocyte antigen (HLA)-A1, -A2, -A11, -A24, -B7, and -B44 of human MHC class I molecules are commonly expressed by the vast majority of the human population [12].

Previously, experiments from other researchers have been done to identify human MHC class I restricted HPV 18 E6/E7-specific CD8 + T cell epitopes. Castellanos et al. [13] used a computer-assisted algorithm to identify (HLA)-A*0201 (HLA-A2) binding peptides from HPV18 E6/E7 protein. They found that HPV18 E6aa13-21, and HPV18E7aa88-97 were able to induce peptide-specific cytotoxicity. Rudolf et al. [14] used binding affinities for all possible 9-mer peptides spanning the entire HPV18 E6/E7 protein sequence for multiple HLA class I molecules. They tested the immunogenicity of five E6-derived and one E7-derived peptide with high affinities for HLA-A2 by in vitro immunization with purified human CD8 + T cells. They identified three HPV-18 E6-derived peptides (E6aa40-48, ELTEVFEFA, E6aa36-44, KTVLELTEV, and E6aa13-21, KLPDLCTEL) and the E7-derived peptide (E7aa7-15, TLQDIVLHL) to be highly immunogenic. However, they did not test which of the identified peptides is endogenously processed and presented by tumor cells. Kather et al. [15] identified a HLA-A2-restricted HPV18 E7-specific CD8 + T cell epitope (aa86-94) using CD80 and HLA-A2 transfected Hela cells. More importantly, they showed that this epitope-specific CD8 + T cells exist in tumor infiltrating lymphocytes of a HPV18-positive, HLA-A2-matched cervical cancer patient. Chen et al. [16] used a combination of epitope prediction, enzyme-linked immunosorbent assay (ELISA)-based epitope-HLA complex formation, and DNA immunization in HLA-A11 transgenic mice and identified two HLA-A11-restriced HPV18 E6 (E6aa54-62 and E6aa84-92)-specific CD8 + T cell epitopes. Furthermore, they found these identified peptides could stimulate CD8 + T cells from HPV18 infected cervical cancer patients.

Here, we use human MHC class I transgenic mice so we can characterize the human MHC class I HPV specific CD8 + T cell epitopes. Knowing the human HPV specific CD8 + T cell epitopes is important for future clinical translation. The use of wild type mice would not permit us to identify the human MHC class I HPV specific CD8 + T cell epitopes. Therefore, the employment of these human MHC class I transgenic mice will serve as an important tool for the identification of key human MHC class I restricted HPV-specific CTL epitopes.

Human MHC class I transgenic mice still have murine MHC class I molecules, meaning E6/E7 proteins could predominantly present through these murine MHC class I molecules instead of human MHC class I molecules [17]. Therefore, it is important to seek strategies to abolish the presentation of E6/E7 proteins through murine MHC class I molecules in order to reliably use these human MHC class I transgenic mice for the identification of human MHC class I restricted CD8 + T cell epitopes. We have previously mutated the murine MHC class I (H-2D^b^) restricted HPV16E7-specific CD8 + T cell epitope (aa49-57) to abolish the presentation of E7 through murine MHC class I molecule [17, 18]. Additionally, we have characterized HPV16 E6 murine MHC class I restricted CD8 + T cell epitopes, and we have characterized human HLA-A2 class I restricted HPV16 E6/E7 epitopes [18]*.* In the current study, we characterized both the murine MHC class I restricted and human MHC class I restricted HPV18 E7-specific and E6-specifc CD8 + T cell epitopes using C57BL/6 mice and the various human MHC class I transgenic mice vaccinated with therapeutic HPV18E7 or HPV18E6 DNA. We found that there is no dominant murine MHC class I restricted HPV18 E7-specific CD8 + T cell epitope in wild-type mice, whereas there is a dominant murine MHC class I restricted HPV18 E6 peptide (amino acid [aa]67–75) specific CD8 + T cell epitope. Thus, in order to characterize human HLA-restricted HPV18 E6 peptide-specific CD8 + T cell epitopes, we use mutated HPV18 E6 DNA to abolish the presentation of E6 through murine MHC class molecules. We have used the HPV18 E7 and mutated E6 to identify human MHC class I restricted CD8 + T cell epitopes using various human MHC class I transgenic mice.

In addition, we have created an HPV18 E7/E6-expressing spontaneous cervicovaginal adenosquamous carcinoma using these HPV18 E7/mutated E6 oncogenic proteins alongside plasmids encoding constitutively active AKT, cMyc, and SB100. AKT (Protein kinase B) is a part of the PI3K/AKT pathway. Mutations in this pathway, such as constitutively active AKT, is a common somatic mutation in HPV-associated cervical cancers [19]. cMyc is an often upregulated gene found in cells infected with HPV, and it is commonly associated with cancer [20–24]. SB100 (sleeping beauty transposase) induces random integration into DNA [25, 26] and helps with the random HPVE6/E7 oncogene integration that is seen in cervical cancer. In the current study, we used human MHC class I transgenic mice to comprehensively characterize these human MHC class I-restricted HPV18 E6/E7-specific CD8 + T cell epitopes. We developed a HPV18 E6/E7-expressing, spontaneous cervical tumor model in HLA-A2 transgenic C57BL/6 mouse. The translational potential of our studies are discussed.

## Materials and methods

### Mice

5–8 weeks old female wild-type, or HLA class I (HLA-A1, A11, A24, B7 and B44) transgenic C57BL/6 mice were purchased from Taconic Biosciences (Germantown, NY). HLA-A^*^0201/D^d^ (AAD) transgenic mice with a C57BL/6 background [27] were kindly provided by Victor Engelhard at the University of Virginia Health Sciences Center and maintained at Johns Hopkins University School of Medicine animal facility. All procedures were performed according to approved protocols and in accordance with recommendations for the proper use and care of laboratory animals.

### Peptides, antibodies and other reagents

HPV18 E6 and E7 overlapping peptides (15 amino acid long and overlapped by 10 amino acids) spanning the full length of HPV18 E6 and E7 protein were synthesized by GenScript (Piscataway, NJ, USA). HPV18 E7aa6-14, ATLQDIVLH, HPV18 E7aa7-15, TLQDIVLHL, HPV18 E6aa24-33, SLQDIEITCV, HPV18 E6aa25-32, LQDIEITC, HPV18 E6aa26-34, QDIEITCVY, HPV18 E6aa84-92, SVYGDTLEK, HPV18 E6aa85-93, VYGDTLEKL, HPV18 E6aa97-104, GLYNLLIR, HPV18 E6aa98-15, LYNLLRC, and HPV18 E6aa97-105, GLYNLLIRC peptides were also synthesized by GenScript. All the peptides were synthesized at a purity of ≥ 80%. PE-conjugated anti-mouse CD8a (clone 53.6.7), FITC-conjugated anti-mouse IFN-γ (clone XMG1.2) antibodies were purchased from Biolegend (San Diego, CA, USA). GolgiPlug, FITC-conjugated anti-human HLA-A2 (clone BB7.2) and PE-conjugated anti-mouse H-2D^d^ (clone 34-5-8S) antibodies were purchased from BD Pharmingen (San Diego, CA). FITC-conjugated anti-mouse CD45 (clone 30-F11) was purchased from Biolegend (San Diego, CA, USA). Recombinant murine IL-2 was purchased from R&D Systems (Minneapolis, MN, USA). Lipofectamine 2000 was purchased from Invitrogen (Waltham, MA, USA). Puromycin was purchased from Gibco (Waltham, MA, USA). Annexin V-PE Apoptosis detection kit was purchased from BD Pharmingen (San Diego, CA, USA). Purified anti-mouse CD3 monoclonal antibody (clone 17A2) was purchased from Bio X Cell (West Lebanon, NH, USA). RBC lysis buffer was purchased from Cell Signalling Technology (Danvers, MA, USA).

### Cells

The C1R cell line is an Epstein-Barr virus-transformed B-cell line that has lost most of its HLA class I alleles, expressing only Cw0401 and trace amounts of B3503 [28]. The C1R murine MHC class I transfectants C1R/D^b^ and C1R/K^b^ were kindly provided by Dr. Michael Edidin (Johns Hopkins University, Baltimore, MD.). Generation of C1R cells expressing HLA-A^*^0201/D^d^ (AAD) has been described previously [29]. The T2-K^b^ cell line, a murine MHC class I transfectant of T2 (174 × CEM.T2) cells that are deficient in TAPs (Transporter associated with antigen processing), was kindly provided by Dr. Jonathan Schneck (Johns Hopkins University). The T2-D^b^ cell line, a murine MHC class I transfectant of T2, and T2-A11 cell line, a human MHC class I transfectant of T2, were kindly provided by Dr. Elizabeth Jaffee (Johns Hopkins University). The generation of TC-1 has been described previously [30]. Establishment of a TC-1 cell line expressing HLA-A^*^0201/D^d^ (TC-1/AAD) has been described previously [17]. These cell lines were cultured in complete RPMI-1640 medium (supplemented with 2 mM glutamine, 1 mM sodium pyruvate, 100 U/ml penicillin, 100 μg/ml streptomycin, 5 × 10^−5^ M β-mercaptoethanol, and 10% fetal bovine serum). HEK 293 cells were purchased from ATCC (Manassas, VA, USA). The generation of HEK 293 cells expressing HLA-A^*^0201/D^d^ (293-AAD) has been described previously [31]. The HPV18 positive human cervical cancer cell line, Hela cell, was purchased from ATCC. Hela cell expressing HLA-A^*^0201/D^d^ (Hela-AAD), was established using the same protocol as described [9] and were selected with puromycin (3 μg/ml). The purpose of using 293-AAD cells is that we are attempting to determine if epitope is processed endogenously by human cells and presented by HLA-A2. However, the T cells we use are from HLA-A2(AAD) transgenic mice. Within the AAD construct, the α1 and α2 domain of the HLA-A2 are derived from human, whereas the α3 domain of the AAD molecule is derived from murine *H-2D*^*d*^ in order to bind with the co-receptor CD8 molecule on murine CD8 + T cells. Furthermore, 293-AAD cells can be easily transfected. The expression of AAD by 293-AAD cells or Hela-AAD cells was confirmed by flow cytometry analysis using antibodies against HLA-A2 and H-2D^d^. HEK 293, 293-AAD, Hela and Hela-AAD cells were cultured in DMEM medium containing 2 mM glutamine, 1 mM sodium pyruvate, 100 IU ml^−1^ penicillin, 100 μg ml^−1^ streptomycin and 10% FBS.

### Plasmids

The generation of pKT2/CLP-AKT plasmid [32] and pCMV(CAT)T7-SB100 plasmid [33] has been described previously. These plasmids were purchased from Addgene. The generation of Pkt2-cMyc has been described previously [31]. To generate Pkt2-Luc-T2a-HPV18E7E6(del D70), 18E6 (delD70) was first amplified via PCR using the Pkt2-LucHPV18E7E6 [34] template and the following set of primers: 5′-CTGGCTCGAGGAGGGAAGGGGAAGCCTGCT-3′, 5′-GCTCCCGGATTCTGCTGTAGAAGATACACTTGTGGCAAGCGGCG-3′, 5′-CGCCGCTTGCCACAAGTGTATCTTCTACAGCAGAATCCGGGAGC-3′, AND 5′-AAACCAGCTAGCTGGTTATTACACCTGGGTCTC-3′. The amplified PCR product was then cloned into the Xho/bstX1 sites of a Pkt2-LucHPV18E7E6. Plasmid construct was confirmed using DNA sequencing and the DNA was prepared using an endotoxin-free kit (QIAGEN, Valencia, CA, USA).

### Vaccines

The development of pBI-11, a DNA vaccine encoding the E6 and E7 proteins from HPV16 and HPV18, was described previously [9]. TA-HPV is a recombinant vaccinia virus expressing HPV16/18-E6/E7, and it has been described previously [35]. To generate pcDNA3-CRTHPV18E7, the HPV18-E7 sequence was synthesized by GenScript and cloned into the EcoRI and HindIII sites of pcDNA3-CRT. The construction of pcDNA3-CRT has been described previously [8]. To generate pcDNA3-CRT/18E6, HPV18E6 was cloned from pcDNA3-HPV18E6 [29] into pcDNA3-CRT by EcoRI/HindIII.

To abolish HPV18-E6aa67-75 (KCIDFYSRI) epitope presentation in the H-2^b^ MHC class I mice, the amino acid 70 (aspartate) of HPV18E6 was deleted to form HPV18E6(delD70) DNA. To generate pcDNA3-CRT/18E6 (delD70), 18E6 (delD70) was first amplified via PCR using the pcDNA3-CRT/18E6 template and the following set of primers: 5′-AAAGAATTCATGGCGCGCTTTGAGGATCC-3′, 5′-GCTGCATGCCATAAATGTATATTTTATTCTAGAATTAGAGAA-3′, 5′-TTCTCTAATTCTAGAATAAAATATACATTTATGGCATGCAGC-3′, AND 5′-CCCAAGCTTTTATACTTGTGTTTCTCTGC-3′. The amplified PCR product was then cloned into the EcoRI/HindIII sites of a pcDNA3-CRT.

### Vaccination

DNA vaccine was prepared in PBS and administered through intramuscular (IM) injection followed by electroporation (EP) using an electro Square Porator (ECM 830, BTX, Holliston, MA, USA). TA-HPV vaccinia virus was administered through skin scarification (SS) as described previously [11]. For vaccination experiments, female mice (5 per group) were vaccinated with 20 μg/mouse of codon optimized pcDNA3-CRT/HPV18E7 DNA, pcDNA-CRT/HPV18E6 DNA, pcDNA-CRT/HPV18E6(delD70) DNA on day 0 through IM injection followed by EP. The mice were boosted once with the same dose and regime on day 7. Then mice were further boosted with 5 × 10^5^ pfu/mouse of TA-HPV vaccinia vaccine through SS on the tail on day 14.

### Prediction of HLA class I restricted potential HPV18 E6/E7 CD8 + T cell epitopes with algorithm

For the prediction of potential HLA class I restricted, HPV18 E6/E7-specific CD8 + T cell epitopes, the algorithm NetMHCpan-4.1 was used [36]. NetMHCpan 4.1 was established based on a combination of more than 850,000 quantitative binding affinity and mass-spectrometry eluted ligands peptides using artificial neural networks. Peptides with lengths of 8–14 amino acids were predicted, and the highest percentile rank and binding affinity score were used to select the peptides to be tested. Strong binding peptides were determined as peptides with a rank threshold less than 0.5 and peptides with a rank threshold between 0.5 and 2.0 as weak binding peptides.

### Intracellular cytokine staining and flow cytometry analysis

To detect HPV18 E6 or E7-specific CD8^+^ T cell responses by IFN-γ intracellular staining, spleens from vaccinated wild type or HLA class I transgenic C57BL/6 mice were harvested 12 days after last vaccination, and minced with a 10 ml syringe plunger against cell strainer (70 μM). Red blood cells were lysed with RBC lysis buffer. The cells were washed once with complete RPMI 1640 medium and resuspended in completed RPMI 1640 medium. 5 × 10^6^ of the prepared splenocytes were plated into each well of 24-well plate and stimulated with either HPV18 E6 or E7 overlapping peptides (5 μg/ml) or HPV18 E6 or E7 short peptides (1 µg/ml) at the presence of GolgiPlug (BD Pharmingen, San Diego, CA, USA) at 37 °C overnight. When HPV18 E6 or E7 peptide-pulsed C1R or T2 MHC class I transfectants were used, these transfectants were first pulsed with indicated HPV18 E6 or E7 peptides (5 μg/ml) at 37 °C for 3 h. These HPV18 E6 or E7 peptide pulsed cells were then washed with RPMI-1640 medium containing 10% FBS three times and co-cultured with splenocytes at the ratio of 25 to 1 (splenocytes to HPV 18 E6 or E7 peptide pulsed cells) at the presence of GolgiPlug at 37 °C overnight. The stimulated splenocytes were stained with PE-conjugated anti-mouse CD8a followed by fixation and permeabilization using the Cytofix/Cytoperm kit according to the manufacturer’s instructions (eBioscience, San Diego, CA, USA). Intracellular IFN-γ was stained with FITC-conjugated anti-mouse IFN-γ. The cells were acquired with FACSCalibur flow cytometer and data were analyzed with CellQuest Pro software.

### Generation of an HLA-A2-restricted HPV18 E7 peptide (aa7-15), or HPV18E6 peptide (aa97-105)-specific CD8 + T cell line

Spleens from HLA-A2 (AAD) transgenic C57BL/6 mice vaccinated with pcDNA3-CRT/HPV18 E7, or pcDNA3-CRT/HPV18 E6(delD70) DNA vaccine followed by TA-HPV vaccinia virus were harvested 12 days after the last vaccination. Splenocytes were prepared as described above, stimulated for 7 days and restimulated every 7 days thereafter with irradiated, HPV18 E7aa7-15 peptide-pulsed TC-1/AAD cells (for HPV18 E7 peptide(aa7-15)-specific CD8 + T cells), or with irradiated, HPV18 E6 peptide (aa97-105)-pulsed TC-1/AAD cells (for HPV18 E6 peptide (aa97-105)-specific CD8 + T cells), at the presence of recombinant murine IL-2 using methods similar to what we described previously [37].

### Detection of cytotoxicity of HPV18 E6/E7 peptide-loaded tumor cell by HPV18 E6 peptide (aa97-105), or HPV 18 E7 peptide (aa7-15)-specific CD8 + T cells

To test whether HPV18 E6 peptide (aa97-105), or HPV 18 E7 peptide (aa7-15)-specific CD8 + T cells can induce cytotoxicity against HPV18 E6 peptide (aa97-105), or HPV 18 E7 peptide (aa7-15)-expressing target cells, we used apoptosis of target cells as readout of cytotoxicity. Specifically, 1 × 10^6^ of TC-1/AAD cells were seeded into each well of 6-well plate, pulsed with either HPV18 E6 peptide (aa97-105), or HPV 18 E7 peptide (aa7-15) (5 μg/ml) overnight. The cells were then washed three times with RPMI 1640 medium containing 10% of FBS. These cells were then co-cultured with either HPV18 E6 peptide (aa97-105), or HPV 18 E7 peptide (aa7-15)-specific CD8 + T cells in 96-well round-bottom plate at indicated E:T ratio at 37 °C for 4 h. The cells were then harvested, washed with Annexin V binding buffer, and stained with FITC-conjugated anti-mouse CD45 and PE-conjugated Annexin V according to the manufacturer’s protocol. The cells were acquired with FACS Calibur flow cytometer. The data was generated by gating on the CD45 negative TC-1/AAD cells. TC-1/AAD cells cultured without CD8 + T cells were used to determine the background of the apoptotic cells. The specific cytotoxicity was defined as percent of annexin V(+) TC-1/AAD cells/Total TC-1/AAD cells.

### Characterization of the presentation of HPV18 E7/E6 CD8 + T cell epitopes through the HLA-A2 molecule using an activation assay with HPV18 E7/E6 specific CD8 + T cell line

To evaluate the identified HLA-A*0201-restricted HPV18 E7 and E6-specific CD8 + T cell epitope presentation in HLA-A2 expressing human cells, we transfected 293-AAD cells with either pcDNA3-CRT/HPV18E7 or pBI-11 using Lipofectamine 2000 according to the manufacturer’s protocol. The transfected 293-AAD cells were collected 24 h later, and co-cultured with either HPV18 E7aa7-15 peptide-specific CD8 + T cells (1:1 ratio) in a 96-well round-bottom plate in the presence of Brefeldin A (5 μg/ml) for 20 to 24 h. Similarly, 293-AAD cells were transfected with either pcDNA3-CRT/18E6(delD70), or pBI-11 using Lipofectamine 2000. The transfected cells were collected 24 h later and co-cultured with HPV18E6aa97-105 peptide-specific CD8 + T cells in the presence of Brefeldin A for 20–24 h. To test whether Hela-AAD cells present HPV18 E7 peptide (aa7-15) and/or HPV18 E6 peptide (aa97-105), Hela-AAD, or Hela cells were co-cultured with HPV18E6 peptide (aa97-105)-specific CD8 + T cells in the presence of Brefeldin A in 96-well round-bottom plate for 20–24 h. The presentation of HLA-A*0201-restricted HPV18 E7 CD8 + T cell epitope, HPV18 E7 peptide (aa7-15), or HLA-A*0201-restricted HPV18 E6 CD8 + T cell epitope, HPV18 E6 peptide (aa97-105) was analyzed by the detection of either HPV-18 E7 peptide (aa7-15) or HPV 18 E6 peptide (aa97-105)-specific CD8 + T cell activation using intracellular IFN-γ flow cytometry analysis.

### Establishment of spontaneous cervicovaginal HPV18 E6/E7-expressing tumor model

To establish HPV18 E6E7-expressing cervicovaginal tumor model using AKT and cMyc oncogenes, 5–8 week old female HLA-A2 (AAD) transgenic mice were injected with anti-mouse CD3 monoclonal antibody ( 200 μg/mouse) through intraperitoneal injection for three continuous days. One day after the last injection, plasmids encoding Pkt2-Luc-T2a-HPV18E7E6(del D70), pKT2/CLP-AKT, Pkt2-cMyc and pCMV(CAT)T7-SB100 (10 µg/plasmid, 30 µl/injection) were injected into cervicovaginal area followed by electroporation with an Electro Square Porator as described previously [34]. Anti-mouse CD3 monoclonal antibody treatment was maintained once weekly. Tumor growth was monitored using bioluminescence imaging (Xenogen IVIS spectrum bioluminescence imaging series 2000, Alameda, CA, USA) and gross inspection. Tumor-bearing mice were sacrificed when either the tumor diameter was greater than 15 mm or the mouse body weight was reduced by 10% compared with age-matched untreated control mice as described previously [31].

### Histology

Spontaneously formed cervicovaginal tumors were surgically removed and placed into 10% buffered formalin phosphate. The tumor tissues were then paraffin embedded and hematoxylin and eosin (H&E) staining were performed. The histology slides were reviewed by two board-certified gynecologic pathologists (Deyin Xing and TC Wu) from the Department of Pathology in the Johns Hopkins University School of Medicine (Baltimore, MD, USA).

### Statistical analysis

Data were expressed as means ± standard deviations (SD). Prism version 9.3.1 was used to perform the statistical analysis of the data. 2-tailed Student’s *t* test (unpaired T test with Welch’s correction and Mann–Whitney test were used for nonparametric test) was used to compare individual data point. Two-way ANOVA was used to compare cytotoxicity induced by HPV18 E6/E7 peptide-specific CD8 + T cells. A *P* value of less than 0.05 was considered significant.

## Results

### Therapeutic HPV18 E7 DNA vaccination did not elicit any significant CD8 + T cell response in HLA-A1, HLA-24, HLA-B7, HLA-B44 transgenic or wild type C57BL/6 mice, but generated strong human HLA-A2 and HLA-A11 restricted HPV18E7-specific CD8 + T cell immune response.

**Fig. 1:**
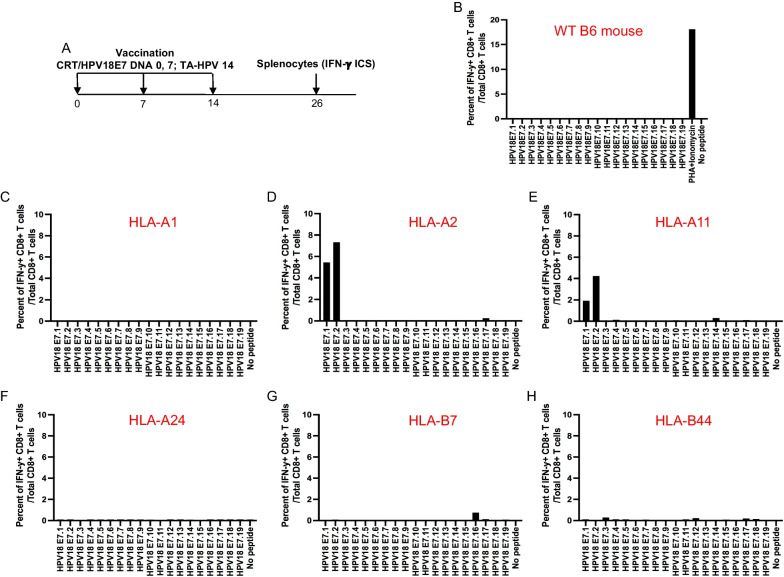
Characterization of HPV18 E7 specific CD8 + T cell immune response in wild type C57BL/6 and different human MHC class I transgenic mice vaccinated with therapeutic HPV vaccine.** A** Schema of the experiment. Briefly, female mice (5 per group) were vaccinated with 20 μg/mouse of pcDNA3-CRT/HPV18E7 DNA vaccine on day 0 through intramuscular injection followed by electroporation. The mice were boosted once with the same dose and regime on day 7. Then mice were further boosted with 5 × 10^5^pfu/mouse of TA-HPV vaccinia vaccine through skin scarification on the tail on day 14. 12 days later, the spleens from the mice were pooled, and the splenocytes were prepared as described in the methods, stimulated with 15mer HPV18 E7 overlapping peptides that span the full-length of the HPV18 E7 protein at the presence of GolgiPlug overnight. The cells were then stained with PE-conjugated anti-mouse CD8a. After permeabilization and fixation, the cells were stained with FITC-conjugated anti-mouse IFN-γ. The cells were acquired with FACSCalibur flow cytometer and data were analyzed with CellQuest Pro software. HPV18 E7 peptide-specific CD8 + T cells were determined as percent of IFN-γ + CD8 + T cells/total CD 8 + T cells. **B** HPV18 E7-specific CD8 + T cell responses in wild-type C57BL/6 mice after vaccination. **C** HPV18 E7-specific CD8 + T cell responses in HLA-A1 transgenic mice after vaccination. **D** HPV18 E7-specific CD8 + T cell responses in HLA-A2 transgenic mice after vaccination. **E** HPV18 E7-specific CD8 + T cell responses in HLA-A11 transgenic mice after vaccination. **F** HPV18 E7-specific CD8 + T cell responses in HLA-A24 transgenic mice after vaccination. **G** HPV18 E7-specific CD8 + T cell responses in HLA-B7 transgenic mice after vaccination. **H** HPV18 E7-specific CD8 + T cell responses in HLA-B44 transgenic mice after vaccination

Previously, we have shown that vaccination with HPV DNA vaccine, pBI-11, followed by TA-HPV vaccinia virus skin scarification was able to significantly enhance HPV16/18 E6 or E7-specific CD8 + T cell responses in C57BL/6 mice [9, 11]. There is no HPV18 E7-specific, H-2^b^-restricted CD8 + T cell epitope that has been reported in C57BL/6 mice. Therefore, we used the same immunization regimen to investigate HPV18 E7-specific CD8 + T cell responses in female C57BL/6 mice with overlapping HPV18 E7 peptides (15mer overlapped by 10 amino acids) that cover the full length of the E7 protein (Fig. 1A). It has been demonstrated that overlapping peptides that span the full-length of the protein can efficiently stimulate both CD4 + and CD8 + T cells [38]. These overlapping peptides have been used as a standard approach to analyze human CD4 + and CD8 + T cell response in therapeutic HPV vaccine clinical trials [39–41]. As shown in Fig. 1B, wild type C57BL/6 mice do not mount a significant CD8 + T cell response to any 15mer HPV18 E7 overlapping peptides. This suggests that HPV18 E7 protein does not possess a murine H-2 D^b^ or H-2 Kb restricted CD8 + T cell epitope.

To characterize human HLA-restricted CD8 + T cell responses, female human HLA-A1, -A2, -A11, -A24, -B7, or -B44 transgenic mice (5 per group) were vaccinated with the same regimen used on the wild type C57BL/6 mice (Fig. 1A). HPV18 E7-specific CD8 + T cell responses from the vaccinated mice were tested with HPV18 E7 overlapping peptides. HLA-A1, HLA-A24, HLA-B7 and HLA-B44 transgenic mice displayed minimal HPV18 E7-specific CD8 + T cell responses (Fig. 1C, F–H). In contrast, both HLA-A2 and HLA-A11 transgenic mice responded with strong HPV18 E7.1 and E7.2 peptide specific CD8 + T cell populations (Fig. 1D, E).

### Vaccination with HPV18 E7 DNA vaccine confirms the predicted HLA-restricted E7-specific CD8 + T cell epitopes in HLA-A2 and HLA-A11 transgenic mice

**Fig. 2:**
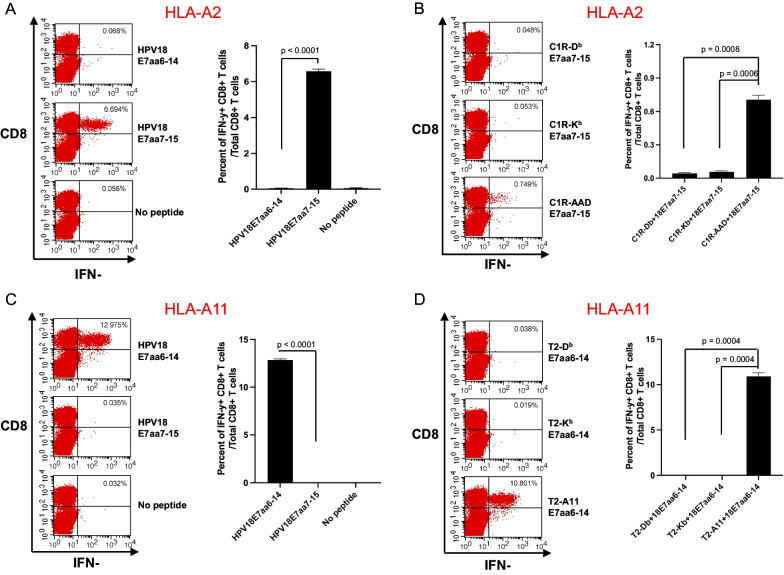
Characterization of HLA-A2 and HLA-A11 restricted HPV18 E7-specific CD8 + T cell responses generated by therapeutic HPV vaccine in human MHC class I transgenic mice. Female HLA-A11 or HLA-A2 transgenic mice (5 per group) were vaccinated as described in Fig. 1. For **A**, **C** 12 days after the final vaccination, the splenocytes from the mice were prepared and stimulated with HPV18 E7.1 peptide, HPV18 E7.2 peptide, HPV18 E7aa6-14 peptide, or HPV18E7aa7-15 peptide at the presence of GolgiPlug overnight. The cells were then stained with PE-conjugated anti-mouse CD8a. After permeabilization and fixation, the cells were stained with FITC-conjugated anti-mouse IFN-γ.The cells were acquired with FACSCalibur flow cytometer and data were analyzed with CellQuest Pro software. For figure B, 12 days after the last vaccination, the splenocytes from the mice were prepared and stimulated with C1R-Db, C1R-Kb, or C1R-AAD cells pulsed with HPV18 E7aa7-15 peptide. For figure D, the splenocytes were stimulated with T2-D^b^, T2-K^b^, or T2-A11 cells pulsed with HPV18 E7aa6-14 peptide at the presence of GolgiPlug overnight. The cells were then stained as described previously. **A** Representative image (left panel) and summary (right panel) of flow cytometry analysis of HPV18 E7-specific CD8 + T cells generated by vaccinated HLA-A2 transgenic mice. **B** Representative image (left panel) and summary (right panel) of flow cytometry analysis following pulsing with C1R-D^b^, C1R-K^b^, or C1R-AAD cells pulsed with HPV18 E7aa7-15. **C** Representative image (left panel) and summary (right panel) of flow cytometry analysis of HPV18 E7-specific CD8 + T cells generated by vaccinated HLA-A11 transgenic mice. **D** Representative image (left panel) and summary (right panel) of flow cytometry analysis following pulsing with T2-D^b^, T2-K^b^ or T2-A11 cells pulsed with HPV18 E7aa6-14

Next, we wanted to determine the HPV18 E7-specific CD8 + T cell epitopes responsible for the immune response observed in Fig. 1. We first used the NetMHCpan-4.1 algorithm to predict candidate HLA restricted HPV18 E7-specific CD8 + T cell epitopes from HPV18 E7 overlapping peptides recognized by CD8 + T cells from the vaccinated mice. As shown in Table 1, for HLA-A2-restricted HPV18 E7-specific CD8 + T cell epitope, peptide (aa7-15) has the highest percentile rank, whereas for HLA-A11-restricted HPV18 E7-specific CD8 + T cell epitope, peptide (aa6-14) has the highest percentile rank. When we ran these peptide sequences with a recently developed algorithm, Prediciton of Immunogenic Epitopes (PRIME2.0) which combines both MHC class I binding affinity together with TCR recognition propensity by Dr. Gfeller at the University of Lausanne, Switzerland (PRIME2.0) [42], similar results were found (data not shown). We then validated these predicted CD8 + T cell epitopes using splenocytes from vaccinated mice. When spenocytes from the vaccinated mice were tested against the predicted epitopes, we found that the HLA-A2 transgenic mice mounted a strong HPV18 E7 peptide (aa7-15)-specific CD8 + T cell immune response (Fig. 2A). To determine the MHC class I restriction element for the CD8 + T cell epitope, we pulsed C1R-D^b^, C1R-K^b^ or C1R-AAD cells with the predicted HPV18 E7 peptide (aa7-15) and used it to stimulate splenocytes from the vaccinated mice. As shown in Fig. 2B, only the HPV18 E7 peptide (aa7-15) pulsed C1R-AAD cells, but not the C1R-D^b^ or -K^b^, demonstrated a strong activation of the HPV18E7 peptide (aa7-15)-specific CD8 + T cell response. The lower reactivity, observed in Fig. 2B, is probably due to the fact that we used a ratio of 25:1 (splenocyte:peptide-pulsed APCs) for activation of HPV antigen-specific CD8 + T cells within the splenocytes. Overall, the data confirms that E7 peptide (aa7-15) was presented through the HLA-A2 molecule.

**Table 1:** Candidate HLA Class I restricted HPV18 E7-specific CTL epitope predicted by NetMHCpan 4.1 algorithm

Overlapping peptide	HLA element	Epitope length	Epitope sequence	Position	Percentile Rank
E7.1 (aa1-15)	A*02:01	8	LQDIVLHL	aa8-15	1.64
		9	**TLQDIVLHL**	aa7-15	0.01
		10	ATLQDIVLHL	aa6-15	0.15
E7.2 (aa6-20)	A*02:01	8	LQDIVLHL	aa8-15	1.64
		9	**TLQDIVLHL**	aa7-15	0.01
		10	ATLQDIVLHL	aa6-15	0.15
E7.1 (aa1-15)	A*11:01	8	TLQDIVLH	aa7-14	12.5
		9	**ATLQDIVLH**	aa6-14	0.33
		10	KATLQDIVLH	aa5-14	1.96
E7.2 (aa6-20)	A*11:01	8	TLQDIVLH	aa7-14	12.5
		9	**ATLQDIVLH**	aa6-14	0.33
		10	ATLQDIVLHL	aa6-15	5.32

Rank threshold for strong binding peptides: 0.500. Rank threshold for weak binding peptides: 2.000. Boldfaced peptides were chosen as predicted epitopes

Next, to confirm the predicted HLA-A11 restricted E7 peptide (aa6-14)-specific epitope (see Table 1), splenocytes from vaccinated mice were stimulated with the predicted epitope peptide, and a strong recongnition by CD8 + T cells from HPV18 E7 vaccinated HLA-A11 transgenic mice (Fig. 2C). To further confirm the MHC class I restriction element, we pulsed T2-D^b^, T2-K^b^ or T2-A11 cells with the predicted HPV18 E7 peptide (aa6-14) and co-cultured with splenocytes from HPV18 E7 vaccinated HLA-A11 transgenic mice*.* As shown in Fig. 2D, only the HPV18 E7 peptide (aa6-14) pulsed T2-A11 cells, but not the T2-K^b^ or -D^b^, demonstrated a strong activation of the HPV18E7 peptide (aa6-14)-specific CD8 + T cell response. This data confirms that the E7 peptide (aa6-14) was presented through the HLA-A11 molecule.

### A single deletion of aspartic acid (D) at location 70 in HPV18E6 DNA abolishes the presentation of HPV18 E6 peptide (aa67-75) by murine MHC class I

**Fig. 3:**
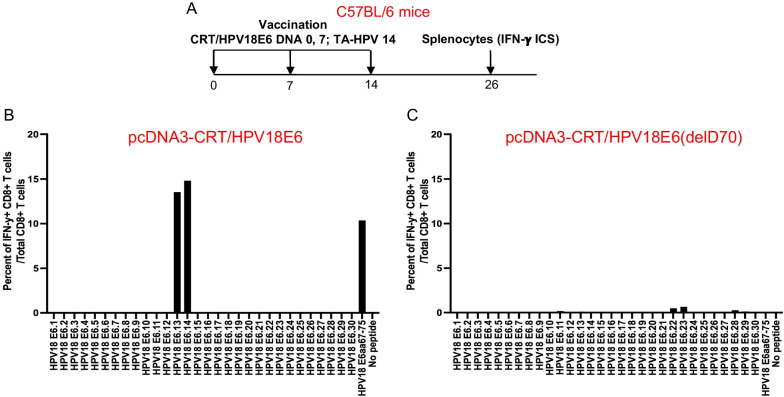
Characterization of HPV18 E6-specific CD8 + T cell immune response in wild type C57BL/6 mice vaccinated with therapeutic HPV vaccine. **A** Schema of the experiment. Briefly, female mice (5 per group) were vaccinated with 20 μg/mouse of pcDNA3-CRT/HPV18E6 DNA or pcDNA3-CRT/HPV18E6(delD70) vaccine on day 0 through intramuscular injection followed by electroporation. The mice were boosted once with the same dose and regime on day 7. Then mice were further boosted with 5 × 10^5^ pfu/mouse of TA-HPV vaccinia vaccine through skin scarification on the tail on day 14. 12 days later, the spleens from the mice were pooled, and the splenocytes were prepared as described in the methods, stimulated with 15mer HPV18 E6 overlapping peptides that span the full-length of the HPV18 E6 protein at the presence of GolgiPlug overnight. The cells were then stained with PE-conjugated anti-mouse CD8a. After permeabilization and fixation, the cells were stained with FITC-conjugated anti-mouse IFN-γ. The cells were acquired with FACSCalibur flow cytometer and data were analyzed with CellQuest Pro software. HPV18 E6 peptide-specific CD8 + T cells were determined as percent of IFN-γ + CD8 + T cells/total CD 8 + T cells. **B** HPV18 E6-specific CD8 + T cell responses in wild-type C57BL/6 mice after vaccination with DNA encoding CRT linked to wild-type HPV18 E6. **C** HPV18 E6-specific CD8 + T cell responses in wild-type C57BL/6 mice after vaccination with DNA encoding CRT linked to mutant HPV18 E6(delD70)

We have previously shown that when a C57BL/6 mouse is vaccinated with HPV18 E6, a strong HPV18 E6aa67-75 peptide-specific CD8 + T cell response was observed [29]. To investigate whether other HPV18 E6-specific CD8 + T cell responses exist, we analyzed HPV18 E6-specific CD8 + T cell responses after vaccination using overlapping peptides covering the full-length of HPV18 E6 protein. Female C57BL/6 mice (5 per group) were vaccinated as described in Fig. 3A. The splenocytes from the vaccinated mice were prepared and stimulated with 15mer HPV18 E6 overlapping peptides that span the full-length of the HPV18 E6 protein. Flow cytometry analysis was then performed to determine HPV18 E6 peptide-specific CD8 + T cells. Consistent with our previous results, the mice vaccinated with wild-type HPV18E6 DNA predominantly mounted a murine H-2D^b^ restricted HPV18 E6 peptide (aa67-75)-specific CD8 + T cell response; no other significant HPV18 E6 peptide-specific CD8 + T cell response was observed (Fig. 3B). In order to eliminate the presentation of the E6 peptide through the murine MHC class I molecule, we generated a mutated HPV18 E6 by deleting aspartic acid (D) at location 70 (HPV18 E6(delD70)). We then vaccinated C57BL/6 mice with the same regimen using DNA encoding CRT linked to the mutant HPV18E6(delD70). The mice vaccinated with the mutant HPV18E6(delD70) DNA were unable to mount any significant HPV18 E6 peptide (aa67-75)-specific CD8 + T cell mediated immune response, and no other HPV18 E6 peptide-specific CD8 + t cell response was observed (Fig. 3C).

### Vaccination with HPV18E6(delD70) DNA generates E6-specific CD8 + T cell mediated immune response in human MHC class I HLA-A2, -A11, A24, and B-44 transgenic mice, but not HLA-A1 or HLA-B7 transgenic mice

**Fig. 4:**
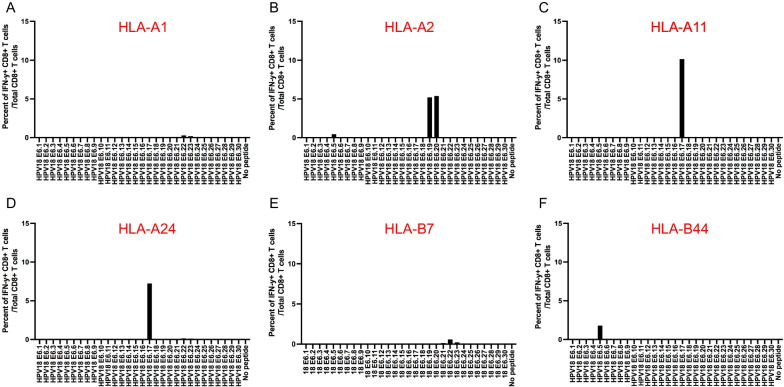
Characterization of HPV18 E6-specific CD8 + T cell immune response in different human MHC class I transgenic mice vaccinated with therapeutic HPV vaccine. Briefly, female mice (5 per group) were vaccinated with 20 μg/mouse of pcDNA3-CRT/HPV18E6(delD70) DNA vaccine on day 0 through intramuscular injection followed by electroporation. The mice were boosted once with the same dose and regime on day 7. Then mice were further boosted with 5 × 10^5^ pfu/mouse of TA-HPV vaccinia vaccine through skin scarification on the tail on day 14. 12 days later, the spleens from the mice were pooled, and the splenocytes were prepared as described in the methods, stimulated with 15mer HPV18 E6 overlapping peptides that span the full-length of the HPV18 E6 protein at the presence of GolgiPlug overnight. The cells were then stained with PE-conjugated anti-mouse CD8a. After permeabilization and fixation, the cells were stained with FITC-conjugated anti-mouse IFN-γ. The cells were acquired with FACSCalibur flow cytometer and data were analyzed with CellQuest Pro software. HPV18 E6 peptide-specific CD8 + T cells were determined as percent of IFN-γ + CD8 + T cells / total CD 8 + T cells. Bar graph of HPV18 E6-specific CD8 + T cell responses in **A** HLA-A1 **B** HLA-A2 **C** HLA-A11 **D** HLA-A24 **E** HLA-B7 or **F** HLA-B44 transgenic mice after vaccination

Female human HLA-A1, A2, A11, A24, B7, or B44 transgenic C57BL/6 mice (5 per group) were vaccinated with pcDNA3-CRT/18E6(delD70) DNA vaccine followed by TA-HPV as described previously in Fig. 3A. The splenocytes from the different human MHC class I transgenic mice were stimulated with 15mer HPV18 E6 overlapping peptides that span the full-length of the HPV18 E6 protein followed by flow cytometry analysis to determine HPV18 E6 peptide-specific CD8 + T cells. HLA-A1 transgenic mice did not mount significant HPV18 E6-specific CD8 + T cell responses to any HPV18 E6 peptide (Fig. 4A, E). We did observe a small peak response for B7-restricted responses to E6.22 and E7.16; however, we could not find any predicted short peptides that could be recognized by CD8 + T cells within these peptides. This might be due to the selection of the short peptides, and peptides of different lengths likely need to be further investigated in the future for B7-restriction. In comparison, HLA-A2 transgenic mice mounted HPV18 E6.19 and E6.20 peptide specific CD8 + T cell responses and a minor E6.5 peptide specific CD8 + T cell response (Fig. 4B). Furthermore, HLA-A11 and HLA-A24 transgenic mice demonstrated a HPV18 E6.17 peptide specific CD8 + T cell response (Fig. 4C, D). Finally, HLA-B44 transgenic mice mounted an HPV18 E6.5 peptide specific CD8 + T cell response (Fig. 4F).

### The predicted HLA-restricted E6-specific CD8 + T cell epitopes in HLA-A2, -A11, -A24, and –B44 transgenic mice were confirmed by activation assays

**Fig. 5:**
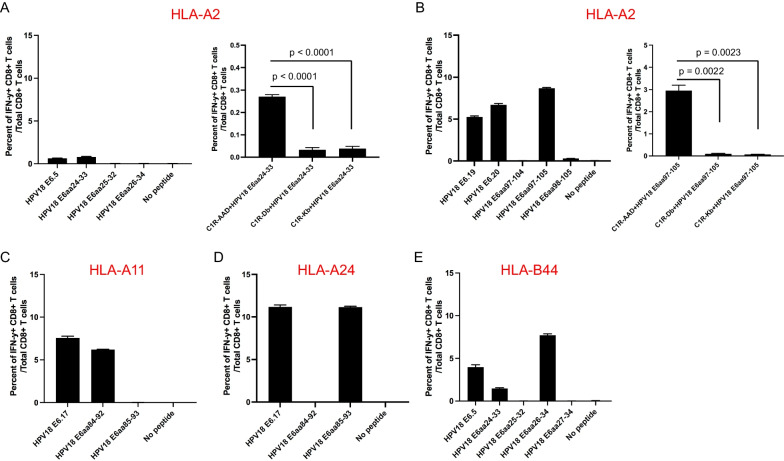
Characterization of HLA-A2, HLA-A11, HLA-24, and HLA-B44 restricted HPV18 E6-specific CD8 + T cell responses generated by therapeutic HPV vaccine in different human MHC class I transgenic mice. Female human MHC class I transgenic mice (5 per group) were vaccinated and cells were prepared as described in Fig. 4. For HLA-A2 mice (**A**, **B**), 12 days after the last vaccination in the HLA-A2 transgenic mice, the splenocytes from the mice were prepared and stimulated with C1R-AAD, C1R-D^b^, or C1R-K^b^ cells pulsed with HPV18 E6 peptide in the presence of GolgiPlug overnight. The cells were then stained, and acquired as described prior. **A.** Flow cytometry analyses of HPV18 E6-specific CD8 + T cells generated by vaccinated HLA-A2 transgenic mice (left hand panel) or following pulsing with C1R-AAD, C1R-D^b^, or C1R-K^b^ cells pulsed with HPV18 E6aa24-33 (right hand panel). **B** Flow cytometry analyses of HPV18 E6-specific CD8 + T cells generated by vaccinated HLA-A2 transgenic mice (left hand panel) or following pulsing with C1R-AAD, C1R-D^b^, or C1R-K^b^ cells pulsed with HPV18 E6aa97-105 (right hand panel). Bar graph of flow cytometry analysis of HPV18 E6-specific CD8 + T cells generated by vaccinated (**C**) HLA-A11 (**D**) HLA-A24 or (**E**) HLA-B44 transgenic mice

To predict potential candidate HLA restricted HPV18 E6-specific epitopes, we once again employed the NetMHCpan-4.1 algorithm (Table 2). HLA-A2 mice are predicted to have two dominant HPV18 E6-specific epitopes: aa24-33 and aa97-105. In addition, HLA-A24 mice are predicted to have an HPV18 E6 peptide (aa85-93) specific CD8 + T cell response. Furthermore, HLA-A11 mice are predicted to mount an HPV18 E6 peptide (aa84-92) specific CD8 + T cell response, and HLA-B44 transgenic mice are predicted to mount an HPV18 E6 peptide (aa26-34) specific CD8 + T cell response (Table 2). To further confirm the predicted HLA restricted HPV18 E6-specific CD8 + T cell epitopes, we stimulated the splenocytes from the vaccinated mice with HPV18 E6 peptides as shown in the left panels of Fig. 5A, B. As predicted by the algorithm, HLA-A2 transgenic mice mounted the strongest CD8 + T cell responses to HPV18E6 peptide (aa97-105) and (aa24-33) epitopes (Fig. 5A, B, left panels). In addition, splenocytes from the vaccinated HLA-A2 transgenic mice were stimulated with C1R-D^b^, C1R-K^b^ or C1R-AAD cells pulsed with HPV18 E6 peptide (aa24-33) or HPV18 E6 peptide (aa97-105). The HPV18 E6 peptide-pulsed C1R-AAD cells were able to activate the splenocytes, suggesting that the E6 (aa24-33 and aa97-105) peptides are presented through the HLA-A2 molecule (Fig. 5A, B, right panels). The same protocol was then repeated with HLA-A11, HLA-A24, and HLA-B44 mice as described above utilizing the respective HPV18 E6 peptides as shown in Fig. 5C–E.

**Table 2:** Candidate HLA class I restricted HPV18 E6-specific CTL epitope predicted by NetMHCpan 4.1 algorithm

Overlapping peptide	HLA element	Epitope length	Epitope sequence	Position	Percentile Rank
E6.5 (aa21-35)	A*02:01	8	SLQDIEIT	aa24-31	12.58
		9	SLQDIEITC	aa24-32	0.46
		10	**SLQDIEITCV**	aa24-33	0.11
E6.19 (aa91-105)	A*02:01	8	GLYNLLIR	aa97-104	11.13
		9	**GLYNLLIRC**	aa97-105	0.57
		10	KLTNTGLYNL	aa92-101	0.99
E6.20 (aa96-110)	A*02:01	8	GLYNLLIR	aa97-104	11.13
		9	**GLYNLLIRC**	aa97-105	0.57
		10	GLYNLLIRCL	aa97-106	0.59
E6.17(aa81-95)	A*11:01	8	VYGDTLEK	aa85-92	3.69
		9	SVYGDTLEK	aa84-92	0.002
		10	DSVYGDTLEK	aa83-92	0.51
E6.17(aa81-95)	A*24:02	8	YGDTLEKL	aa86-93	2.95
		9	VYGDTLEKL	aa85-93	0.03
		10	SVYGDTLEKL	aa84-93	0.61
E6.5 (aa21-35)	B*40:02	8	QDIEITCV	aa26-33	2.76
		9	**QDIEITCVY**	aa26-34	4.19
		10	QDIEITCVYC	aa26-35	14.19

Rank threshold for strong binding peptides: 0.500. Rank threshold for weak binding peptides: 2.000. Boldfaced peptides were chosen as predicted epitopes

Activation of splenocytes from the vaccinated transgenic mice using different HPV18 E6 peptides demonstrated that HPV18 E6 peptide (aa84-92) may be a dominant CD8 + T cell epitope for HLA-A11 transgenic mice, which is consistent with the previous findings [16] (Fig. 5C), HPV18 E6 peptide (aa85-93) may be the dominant epitope for HLA-A24 transgenic mice (Fig. 5D), and HPV18 E6 peptide (aa26-34) may be the dominant epitope for HLA-B44 mice (Fig. 5E). For HLA-B44-restricted HPV18 E6.5 15mer peptide-specific CD8 + T cell response, NetMHCPan 4.1 algorithm did not show any strong or weak binders. However, both HPV18 E6 peptide (aa26-34) and HPV18 E6 peptide (aa24-33), which contains the 8mer of HPV18 E6aa26-33, did activate CD8 + T cells (Fig. 5E), while HPV18 E6 peptide (aa26-34) activated significantly more than HPV18 E6 peptide (aa24-33) did (p < 0.0001), suggesting that amino acid position 34 is important for this CD8 + T cell epitope. The identification of a non-MHC class I binder as the HPV E6-specific CTL epitope highlighted that the employment of algorithm for the prediction of the CTL epitope may potentially miss some real CD8 + T cell epitoipes. Additionally, it highlighted the advantage of overlapping peptides for the screening of peptide-specific CD8 + T cell response induced by vaccination.

### HLA-A2 restricted, HPV18 E7 peptide (aa7-15)- and HPV18 E6 peptide (aa97-105)-specific CD8 + T cells are able to induce apoptosis of the specific peptide-loaded target cells and these CTL epitopes are endogenously processed by HPV18 positive Hela-AAD cells

**Fig. 6:**
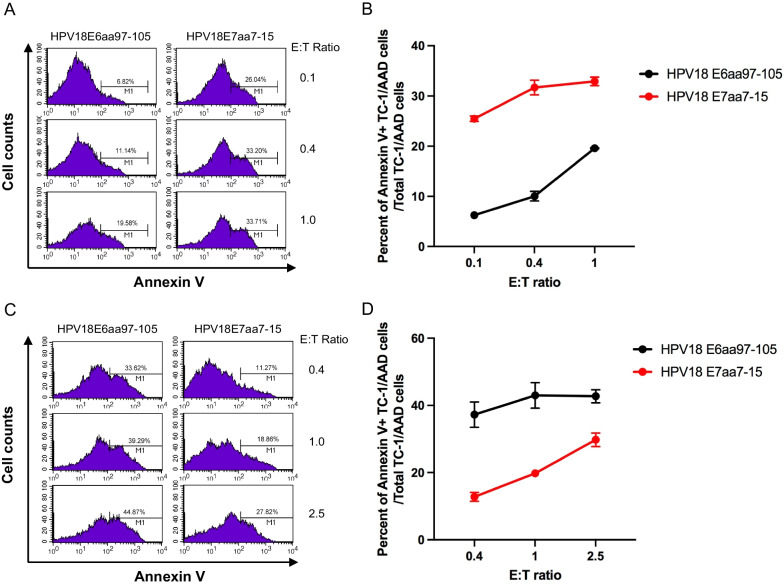
Characterization of apoptosis of HPV18 E6 peptide (aa97-105), or HPV 18 E7 peptide pulsed TC-1/AAD cells induced by HPV18 E6 peptide (aa97-105), or HPV 18 E7 peptide (aa7-15)-specific CD8 + T cells. To detect the apoptosis induced by HLA-A2-restricted HPV18 E6 peptide (aa97-105), or HPV 18 E7 peptide (aa7-15)-specific CD8 + T cells, TC-1/AAD cells were pulsed with either HPV18 E6aa97-105, or HPV 18 E7aa7-15 peptide. After wash, these cells were co-cultured with either HPV18 E6 peptide (aa97-105), or HPV 18 E7 peptide (aa7-15)-specific CD8 + T cells at indicated E:T ratio for 4 h at 37 °C. The cells were then stained with FITC-conjugated anti-mouse CD45 and PE-conjugated Annexin V in annexin binding buffer followed by flow cytometry analysis. Data were analyzed on CD45 negative cells. **A** Flow cytometry analysis of apoptosis of HPV18 E7 peptide (aa7-15) or HPV18 E6 peptide (aa97-105) pulsed TC-1/AAD cells by HPV18 E7 peptide (aa7-15)-specific CD8 + T cells. **B** Summary of flow cytometry analysis of apoptosis of TC-1/AAD cells by HPV18 E7 peptide (aa7-15)-specific CD8 + T cells. **C** Flow cytometry analysis of apoptosis of HPV18 E7 peptide (aa7-15) or HPV18 E6 peptide (aa97-105) pulsed TC-1/AAD cells by HPV18 E6 peptide (aa97-105)-specific CD8 + T cells. **D** Summary of flow cytometry analysis of apoptosis of TC-1/AAD cells by HPV18 E6 peptide (aa97-105)-specific CD8 + T cells

An HLA-A2 restricted HPV18 E7 peptide (aa7-15)-specific CD8 + T cell line and an HLA-A2 restricted HPV18 E6 peptide (aa97-105)-specific CD8 + T cell line were established, as described in the Materials and Methods. We showed that these HPV18 E7 (Fig. 6A, B) or HPV18 E6 (Fig. 6C, D) peptide-specific CD8 + T cells were able to induce significantly higher (p < 0.001) apoptosis of the relevant peptide-loaded target cells compared to target cells loaded with irrelevant peptides. These data demonstrated that these peptide-specific CD8 + T cells are very specific in recognizing and killing the target cell loaded with the specific peptide but not the irrelevant peptide.

**Fig. 7:**
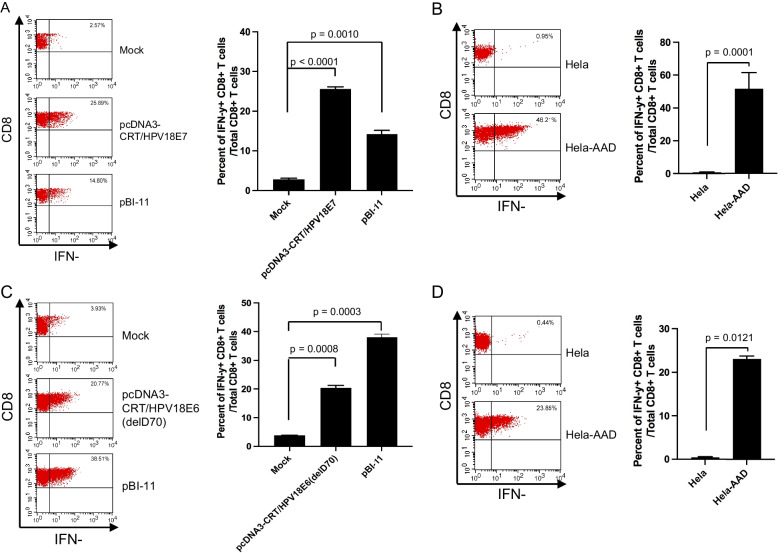
Activation of HPV18E7- or HPV18E6-specific CD8 + T cells by TC-1/AAD cells transfected with either HPV18 E7 or HPV18E6(delD70) DNA, or by HPV18 positive human cervical cancer cell line, Hela-A2 (AAD). HLA-A2 restricted HPV18 E7 peptide (aa7-15) specific CD8 + T cell line was established using splenocytes derived from HLA-A2(AAD) transgenic mice vaccinated with pcDNA3-CRT/HPV18E7 DNA and boosted with TA-HPV vaccinia. The splenocytes were incubated with irradiated TC-1/AAD cells pulsed with HPV18 E7 peptide (aa7-15) in the presence of murine IL-2. An HLA-A2 restricted HPV18 E6 peptide (aa97-105) specific CD8 + T cell line was established using splenocytes derived from HLA-A2(AAD) transgenic mice vaccinated with pcDNA-3CRT/HPV18E6(delD70) DNA and boosted with TA-HPV vaccinia. The splenocytes were incubated with irradiated TC-1/AAD cells pulsed with HPV18 E6 peptide (aa97-105) in the presence of murine IL-2. For the activation assays using 293-AAD cells, cells were transfected with either pcDNA3-CRT/HPV18E7, or pcDNA3-CRT/HPV18E6(delD70), or pBI-11 using lipofectamine 2000. The transfected cells were then co-cultured with HPV18E7 or HPV18E6 specific CD8 + T cells at the presence of GolgiPlug overnight. For the activation assays using Hela-AAD cells, Hela-AAD, or Hela cells were cocultured with HPV18 E7 or HPV18 E6 specific CD8 + T cells at the presence of GolgiPlug overnight. The cells were harvested, stained with PE-conjugated anti-mouse CD8a. After permeabilization and fixation, the cells were stained with FITC-conjugated anti-mouse IFN-γ. The cells were acquired with FACSCalibur flow cytometer and data were analyzed with CellQuest Pro software. **A** Representative image (left panel) and summary (right panel) of flow cytometry data from HPV18 E7 peptide (aa7-15)-specific CD8 + T cell activation by transfected 293-AAD cells. **B** Representative image (left panel) and summary (right panel) of flow cytometry data from HPV18 E7peptide(aa7-15)-specific CD8 + T cell activation by Hela-AAD cells. **C** Representative image (left panel) and summary (right panel) of flow cytometry data from HPV18 E6 peptide (aa97-105)-specific CD8 + T cell activation by transfected 293-AAD cells. **D.** Representative image (left panel) and summary (right panel) of flow cytometry data from HPV18 E6 peptide (aa97-105)-specific CD8 + T cell activation by Hela-AAD cells

Previously, Rudolf et al. [14] found the E7-derived peptide (E7aa7-15, TLQDIVLHL) to be highly immunogenic. However, they did not test which of the identified peptides is endogenously processed and presented by tumor cells. We first used 293-AAD cells to test whether these two HLA-A2-restricted HPV18 E6/E7 CD8 + T cell epitopes could be processed and presented by human cells. We transfected 293-AAD cells with either mock DNA, pcDNA3-CRT/HPV18E7 DNA, pcDNA3-CRT/HPV18E6(delD70) DNA or pBI-11 DNA using lipofectamine 2000. The transfected cells were then co-cultured with either the HPV18 E7 peptide (aa7-15)-specific or the HPV18 E6 peptide (aa97-105)-specific CD8 + T cell line at the presence of GolgiPlug overnight. Flow cytometry analysis was performed to determine the activation of CD8 + T cells by transfected 293-AAD cells. As shown in Fig. 7A, cells transfected with pcDNA3-CRT/HPV18E7 or pBI-11 were able to activate HPV18 E7 peptide (aa7-15)-specific CD8 + T cells compared to cells transfected with control DNA. Of note, HPV18E7 is codon optimized in both pcDNA3-CRT/18E7 and pBI-11. Likewise, 293-AAD cells transfected with pcDNA3-CRT/HPV18E6(delD70) or pBI-11 were able to activate HPV18 E6 peptide (aa97-105)-specific CD8 + T cells (Fig. 7C).

It is critical for immunotherapy to prove whether HPV18 positive cancer cells process and present these epitopes. Therefore, we overexpressed HLA-A^*^0201/D^d^ (AAD) on HPV18 positive cervical cancer cell line, Hela, to establish Hela-AAD. We then co-cultured Hela-AAD cells with either HPV18 E7 peptide (aa7-15) or HPV18 E6 peptide (aa97-105)-specific CD8 + T cells. We found that Hela-AAD cells, but not Hela cells, were able to activate HPV18 E7 peptide (aa7-15) (Fig. 7B) or HPV18 E6 peptide (aa97-105) (Fig. 7D)-specific CD8 + T cells. Since only Hela-AAD cells, but not Hela cells, were able to activate these CD8 + T cells, our data suggest that this activation is unlikely due to allo-recognition. Taken together, our data indicate both HPV18 E7 peptide (7–15) and HPV18 E6 peptide (aa97-105) are endogenously processed and presented by HPV18 positive cervical cancer cells.

### Injection of DNA plasmids encoding LucHPV18E7E6(delD70), AKT, cMyc, and SB100 followed by EP can result in the development of adenosquamous carcinoma in the cervicovaginal tract of HLA-A2 transgenic mice

**Fig. 8:**
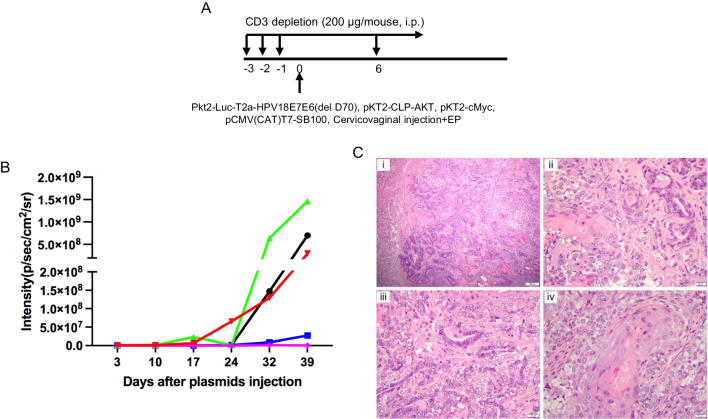
Characterization of spontaneous HPV18E6(delD70)/E7 expressing cervicovaginal tumor in human HLA-A2 transgenic mice. **A** Schematic of the experiment. Briefly, female humanized HLA-A2 transgenic mice (5 per group) received intraperitoneal (ip) injection of anti-CD3 antibody on days-3, -2, -1, then once a week for the duration of the experiment. On day 0, mice were injected with DNA plasmids encoding Luciferase, HPV18 E6(delD70)/E7, AKT, cMyc, and SB100 in the cervicovaginal cavity followed by electroporation (EP). **B** Graph of bioluminescence values of the tumor growth. **C** Histological examination of HPV18E6(delD70)/E7 expressing cervicovaginal tumor. As described in Fig. 7, HPV18E6(delD70)/E7 expressing cervicovaginal tumors were induced in female humanized HLA-A2 transgenic mice (5 per group). Between day 36 and 57, 4 mice were sacrificed and their reproductive tracts were harvested and fixed in formalin for sectioning and histological analysis. Representative images of the histological examination were selected from one mouse. (i) 100 × magnification and (ii) 400 × magnification of a section of the cervicovaginal cavity showing mixed squamous and glandular components. (iii) 400 × magnification of adenocarcinoma in the cervicovaginal tract. (iv) 400 × magnification of areas showing features of squamous cell carcinoma in the cervicovaginal tract

In order to demonstrate that the mutant HPV18 E6(delD70) DNA still remains oncogenic, we set out to induce a spontaneous cervicovaginal carcinoma in HLA-A2 transgenic mice using the mutant HPV18 E6(delD70) DNA. We have previously demonstrated that injection of HPV16E6/E7 with AKT, cMyc, and SB100 following transient CD3 depletion can result in a highly applicable cervicovaginal carcinoma model [34]. Here, to generate a spontaneous HPV18 E6/E7 driven cervicovaginal tumor model, mice were transiently depleted by daily intraperitoneal (IP) injection of antiCD3 antibody once a day for three days, then once a week for the duration of the experiment. Then, DNA plasmids encoding LucHPV18E7E6(delD70), AKT, cMyc, and SB100 were injected into the cervicovaginal tract of HLA-A2 transgenic mice (5 per group) followed by electroporation (Fig. 8A). At least 3 of the 5 mice had significant tumor outgrowth as demonstrated by the increase of the luciferase activity over time (Fig. 8B), as was shown in a statistical analysis (Mann–Whitney Test). Although the p-values were not always significant (for instance, between day 24 and day 10 p = 0.0556), this may be due to the small sample size (n = 5). Because luciferase and HPV18E7/E6(delD70) are on the same gene construct, the luciferase intensity is an indication of the expression of E7/E6(delD70) as well as the tumor growth. In a previous publication [34], we have been able to demonstrate that cells that have luciferase activity also express E6/E7, AKT, and c-Myc, even though AKT and c-Myc are on their own plasmids. In all, our data indicates that the mutation of HPV18 E6(delD70) DNA does not significantly alter the oncogenicity of the oncogene and demonstrates the ability of the mutant HPV18 DNA to generate tumor.

We further examined the histological features of the spontaneous cervicovaginal tumors harvested between days 36 and 57 after plasmid electroporation. The sections showed an infiltrating mass lesion (Fig. 8Ci) with intimately admixed squamous and glandular components (Fig. 8Cii). Some areas predominantly displayed the features of adenocarcinoma (Fig. 8Ciii), while other areas show the morphology of squamous cell carcinoma (Fig. 8Civ). As such, this lesion dominantly displays as an adenosquamous carcinoma that recapitulates the initiation and progression of high-risk HPV related adenosquamous carcinoma in human clinical cases.

## Discussion

In the present study, we characterized both murine and human MHC class I restricted HPV18 E7 and E6 peptide-specific CD8 + T cell epitopes after vaccination with therapeutic HPV18 E6/E7 vaccine. We verified previously reported HLA-A2-restricted HPV18 E7aa7-15 CD8 + T cell epitope (Fig. 2A, B) and HLA-A11-restricted HPV 18 E6aa84-92 CD8 + T cell epitope (Fig. 5C). We also identified a few novel human MHC class I-restricted HPV18 E6/E7-specific CD8 + T cell epitopes, including HLA-A2-restricted HPV18 E6aa97-105, HLA-A2-restricted HPV18 E6aa24-33, HLA-A24-restricted HPV18 E6aa85-93, HLA-B44-restricted HPV18 E6aa26-34 and HLA-A11-resticted HPV18 E7aa6-14 CD8 + T cell epitopes.

As mentioned, HPV18 is a major hrHPV type, along with HPV16. HPV18 has been implicated in a subset of cervical cancers, and HPV18 E6 and E7 are oncogenic (for review see [43, 44]). Identifying the correct HPV18 E6/E7 peptide specific epitopes and their corresponding MHC class I type has significant value. For instance, knowing immunodominant CD8 + T cell epitopes is important for the development of several CD8 + T cell-mediated immunological assays, such as peptide loaded MHC Class I tetramer and intracellular cytokine stains for gamma interferon. In addition, once the specific MHC class I restricted CD9 + T cell epitopes are identified, we can use that information to develop HPV-peptide and/or protein based vaccines (for review see [45]). Furthermore, identifying MHC class I restricted HPV18 E6/E7-specific epitopes also serves as an important foundation for the development of T cell receptor engineered T cell therapy for the control of HPV-associated cancers.

In the current study, we used an approach that combines overlapping peptides and HLA class I peptide binding affinity algorithm to characterize HLA class I-restricted HPV18 E6/E7specific CD8 + T cell epitopes. We first used overlapping peptides (15mer overlapped by 10 amino acids) spanning the full length of HPV18 E6/E7 protein to screen the potential region(s) recognized by HPV18 E6/E7-specific CD8 + T cells since these overlapping peptides have been demonstrated that they can efficiently stimulate both peptide-specific CD4 + and CD8 + T cells [38, 46]. We then used an algorithm, NetMHCpan 4.1 to predict the candidate HPV18 E6/E7 specific CD8 + T cell epitopes (see Fig. 2).

We would like to point out that although the 15mer peptides with 10 overlapping amino acids that we used for these studies will likely cover most of the CD8 + T cell epitopes ranging from 8 to 10 amino acids (the most common length of the epitope), our approach may not be able to detect an epitope that is more than 10 amino acids. However, we did check the peptide with length 11–14 using NetMHCpan 4.1 algorithm, and did not find peptides with higher scores. Once we know the specific peptides from the algorithm, it will be easier to expand the peptide specific CD8 + T cells and characterize their T cell receptors for subsequent development of TCR engineered CD8 + T cells for cancer immunotherapy [47].

Another approach to characterize these MHC class I restricted HPV18 E6/E7-specific epitopes is to use HLA class I peptide binding affinity algorithm, such as NetMHCpan 4.1 to predict the candidate CD8 + T cell epitopes using HPV18 E6 or E7 as whole protein. However, an algorithm as good as NetMHCpan 4.1 can only identify 96.5% of CD8^+^ T cell epitopes, while rejecting 98.5% of non-epitopes ([48]). Although, in general, this approach could potentially be used to identify many of the human MHC class I epitopes (see Additional file 1: Tables S1, S2), it is still possible to miss the low score HLA class I-restricted CTL epitopes. Indeed, as shown in the Additional file 1: Table S2, HLA-B44-restricted HPV18 E6-specific CD8 + T cell epitopes were either weak binder or non-binders. Yet, we are able to identify the HPV18 E6 specific CTL epitope using the overlapping peptide approach. It is of interest in the future to characterize all the algorithm-predicted HPV antigen-specific CTL epitope restricted by different human MHC Class I molecules using the splenocyte from the human MHC Class I transgenic mice vaccinated with effective therapeutic HPV vaccine as described in our system so that we can compare the identified HPV antigen specific CTL epitopes with those identified through the usage of overlapping peptide.

It is important to further validate all of the HLA-restricted CTL epitopes found in mice using human peripheral blood mononuclear cells. Some of our identified CTL epitopes have been reported using human cells previously. For example, Rudolf et al. reported the HLA-A2 restricted E7 peptide (aa7-15, TLQDIVLHL) to be highly immunogenic using human CD8 + T cells [14]. Chen et al. [16] showed that the identified E6 peptide (aa 84-92) could stimulate T-cells to secrete IFNgamma from HPV18-infected patients. Therefore, our data generated from HLA class I transgenic mice correlated well with the data generated using human cells. We intend to validate our findings in PBMCs from HPV18 positive cervical cancer patients when samples become available.

For CD8 + T cell-mediated cancer immunotherapy, it is important to demonstrate that cancer cells do present the epitopes recognized by epitope-specific CD8 + T cells. In the current study, we showed that HPV18 positive cervical cancer cell line, Hela, when HLA-A^*^0201/D^d^ (AAD) was overexpressed, can be recognized by both HLA-A2-restricted HPV18 E6 peptide (aa97-105) and HPV18 E7 peptide (aa7-15)-specific CD8 + T cells, indicating that these two epitopes can be processed and presented by this HPV18 positive cervical cancer cells. Previously, Kather et al. [15] identified a HLA-A2-restricted HPV18 E7-specific CD8 + T cell epitope (aa86-94) using CD80 and HLA-A2 transfected Hela cells.

In the current study, we did not observe this epitope-specific CD8 + T cell response after vaccination of HLA-A2 transgenic mice. It is possible that HPV18 E7 peptide (aa7-15), as described by Rudolf et al. [14], is a dominant epitope. This can be further tested in our system using a HPV18 E7 mutant that abolishes aa7-15 peptide-specific CD8 + T cell responses. We further showed that a candidate therapeutic HPV DNA vaccine, pBI-11, is capable of being endogenously processed and presented by HLA-A2, which is crucial preliminary data to show that the vaccine can be effective in generating HPV18 E6/E7-specific CD8 + T cell-mediated immune responses in humans with HLA-A2 haplotype.

In the current study, we used 6 HLA class I (HLA-A1, A2, A11, A24, B7 and B44) transgenic mouse strain under C57BL/6 background. One issue, as we discussed, is that these mice still have complete murine MHC class I molecules and the processing and presentation of murine MHC class I-restricted CD8 + T cell epitopes could be dominant over human MHC class I-restricted CD8 + T cell epitopes. To overcome this issue, we modified HPV18 E6/E7 sequence to abolish murine MHC class I-restricted CD8 + T cell epitopes. Another approach is using mouse models that lack all murine MHC molecules, such as HLA-A*0201 and HLA-DRB1*0101 transgenic A2.DR1 mice ([49]). This model allows the use of wild-type HPV 18 E6/E7 genes for the characterization of CTL epitopes. Although currently the mouse model that lacks all murine MHC molecules is only available for HLA-A*0201 and HLA-DRB1*0101, it will be interesting to compare the immune responses from AAD mice and A2.DR1 mice in the future and especially for the establishment of spontaneous HPV18 E6/E7-expressing cervical cancer model.

In addition to identifying dominant HLA-restricted HPV18 E6/E7 CD8 + T cell epitopes, we generated a spontaneous adenosquamous carcinoma model using LucHPV18E7E6(delD70), AKT, cMyc, and SB100 in HLA-A2 transgenic mice (Fig. 8). It is not uncommon for adenosquamous carcinoma to develop in cervical cancer, and approximately 10% of cervical carcinomas can display with adenosquamous morphology of mixed malignant squamous and glandular epithelial portions. Furthermore, many of the adenosquamous carcinoma were found to be associated with HPV-18 [50, 51]. Our data demonstrate that the mutated HPV18 E6 is still oncogenic and the single deletion did not significantly impair its function. A major benefit of this model is that it prevents HPV18E6/E7 antigen from presenting via murine MHC class I, but rather the antigen will present via human MHC class I. Furthermore, HPV18 E6/E7-expressing tumor cell line lack of murine MHC class I restricted CD8 + T cell epitopes can be generated from these spontaneously established tumors. These tumor cell lines can be used as transplantable tumor model for studies, such as therapeutic vaccine development and other CD8 + T cell mediated immunotherapy.

However, there are still major restrictions to the model. The model requires CD3 T cell depletion throughout the duration of the experiment. Future studies should better define the amount of time that is needed for tumor outgrowth to occur and how to cease CD3 depletion within that window so that immune cells in the mouse can recover to more typical levels while tumors grow. If we can achieve a model that does not require CD3 T cell depletion, it would better capture the HPV-associated cervical cancer immune environment and would be of more use for studying potential immunotherapies. In addition to immunotherapies, the model could then be used to study molecular intervention that targets E6, E7, AKT, or cMyc, the oncogenes used for tumor induction. Molecular intervention that either directly targeted one of these molecules or targeted a molecule within the downstream pathway of E6/E7/AKT/cMyc could be further explored.

An alternate method to improve upon this model to avoid systemic immunosuppression by CD3 depletion may be to attempt to incorporate gene encoding immunosuppressive molecules that are more typical of cervical cancer into the model. Cervical cancers often express elevated levels of various immunosuppressive molecules, including interleukin (IL) 6 [52, 53], IL10 [53–55], indoleamine-2,3-dioxygenase (IDO) [56–59], programmed death ligand 1 (PD-L1) [60, 61], hypoxia-inducible factor 1 (HIF-1α) [62], vascular endothelial growth factor (VEGF) [63, 64], and transforming growth factor beta (TGF-β) [55, 65]. Further, in addition to binding and inhibiting functions of tumor suppressor proteins p53 and pRB, HPV E6 and E7 can also interact and inhibit expressions of pro-inflammatory cytokines, such as interferon regulatory factor 1 (IRF1) [66] and IRF3 [67], and thereby generate an immunosuppressive TME [68, 69]. Thus, co-transfecting the LucHPV18E7/E6(delD70), AKT, cMyc, and SB100 tumor model with DNA encoding one of these immunosuppressive molecules at a time may result in a cervicovaginal tumor model that does not require CD3 depletion and can incorporate aspects of the tumor microenvironment that is seen clinically.

## Conclusion

In summary, we have successfully identified and characterized various human HLA class I-restricted HPV18 E6 and E7 peptide specific CD8 + T cell epitopes. We have shown that wild type HPV18 E7 cannot be presented through murine MHC class I, whereas wild type HPV18 E6 does have a murine MHC class I CD8 + T cell epitope. HPV18 positive cervical cancer cell line, Hela, expressing chimeric HLA-A2 (AAD) does present both HLA-A2-restricted HPV18 E7 (aa7-15)- and HPV18 E6 (aa97-105)-specific CD8 + T cell epitopes. A candidate therapeutic HPV DNA vaccine, pBI-11 can generate these epitopes in human cells which suggests that such therapeutic HPV DNA vaccine could be used as immunotherapy for patients with HPV18 positive cancers. A mutant HPV18 E6 that had a single deletion at location 70 obliterates the E6 presentation by murine MHC class I and the mutant HPV18E6(delD70) remains oncogenic. At this time, there is still future study that needs to be done in order to improve upon this HPV18 E6/E7 expressing model so that it does not require CD3 depletion. Overall, the identification of these human MHC class I restricted HPV18 E6/E7-specific CD8 + T cell epitopes may have significant future translational potential for either the development of immunological assays or future model development on which immunotherapies or molecular interventions can be tested.

## Supplementary Information

**Additional file 1: Table S1**. Candidate HLA class I restricted HPV18 E7-specific CTL epitope predicted by algorithm as whole HPV18 E6 protein. **Table S2. **Candidate HLA class I restricted HPV18 E6-specific CTL epitope predicted by algorithm as whole HPV18 E6 protein.

## Data Availability

Not applicable.

## Abbreviations

HPV: Human Papillomavirus
hrHPV: High-risk HPVs
CRT: Calreticulin
MHC: Major histocompatibility
HLA: Human leukocyte antigen
aa: Amino acid
IM: Intramuscular
EP: Electroporation
D: Aspartic acid
IP: Daily intraperitoneal
