# Supplementary material for: Identification of human MHC-I HPV18 E6/E7-specific CD8 + T cell epitopes and generation of an HPV18 E6/E7-expressing adenosquamous carcinoma in HLA-A2 transgenic mice
Source: J Biomed Sci. 2022 Oct 12;29:80. doi: 10.1186/s12929-022-00864-5 (PMC9554842; doi:10.1186/s12929-022-00864-5)
Supplement: Supplementary file 1 — Additional file 1: Table S1. Candidate HLA class I restricted HPV18 E7-specific CTL epitope predicted by algorithm as whole HPV18 E6 protein. Table S2. Candidate HLA class I restricted HPV18 E6-specific CTL epitope predicted by algorithm as whole HPV18 E6 protein. [file 12929_2022_864_MOESM1_ESM.docx]

**Table S1: Candidate HLA class I restricted HPV18 E7-specific CTL epitope predicted by algorithm as whole HPV18 E6 protein**

| HLA element | Epitope length | Epitope sequence | Position | Percentile Rank |
| --- | --- | --- | --- | --- |
| A*01:01 | 8 | N/A |  |  |
|  | 9 | EIDGVNHQH | aa40-48 | 0.818 |
|  |  | RAEPQRHTM | aa53-61 | 1.749 |
|  |  | SSADDLRAF | aa78-86 | 1.931 |
|  | 10 | N/A |  |  |
| A*02:01 | 8 | FLNTLSFV | aa90-97 | 1.32 |
|  |  | LQDIVLHL | aa8-15 | 1.635 |
|  | 9 | **TLQDIVLHL** | aa7-15 | 0.01 |
|  |  | FQQLFLNTL | aa86-94 | 0.991 |
|  | 10 | ATLQDIVLHL | aa6-15 | 0.154 |
|  |  | VLHLEPQNEI | aa12-21 | 0.828 |
|  |  | QLFLNTLSFV | aa88-97 | 1.191 |
| A*11:01 | 8 | N/A |  |  |
|  | 9 | **ATLQDIVLH** | aa6-14 | 0.327 |
|  |  | HTMLCMCCK | aa59-67 | 1.216 |
|  | 10 | KATLQDIVLH | aa5-14 | 1.964 |
| A*24:02 | 8 | LFLNTLSF | aa89-96 | 1.105 |
|  | 9 | QLFLNTLSF | aa88-96 | 0.034 |
|  |  | TLQDIVLHL | aa7-15 | 1.601 |
|  |  | FQQLFLNTL | aa86-94 | 1.952 |
|  | 10 | AFQQLFLNTL | aa85-94 | 1.512 |
| B*07:02 | 8 | EPQRHTML | aa55-62 | 0.891 |
|  | 9 | RAEPQRHTM | aa53-61 | 0.188 |
|  |  | GPKATLQDI | aa3-11 | 0.538 |
|  | 10 | RAEPQRHTML | aa53-62 | 0.72 |
|  |  | RRAEPQRHTM | aa52-61 | 0.902 |
|  |  | GPKATLQDIV | aa3-12 | 1.551 |
| B*40:02 | 8 | AEPQRHTM | aa54-61 | 0.401 |
|  |  | NEIPVDLL | aa19-26 | 0.501 |
|  | 9 | AEPQRHTML | aa54-62 | 0.316 |
|  |  | DDLRAFQQL | aa81-89 | 1.044 |
|  |  | CEARIELVV | aa68-76 | 1.1 |
|  | 10 | RAEPQRHTML | aa53-62 | 1.63 |
|  |  | KCEARIELVV | aa67-76 | 1.641 |

Rank threshold for strong binding peptides: 0.500. Rank threshold for weak binding peptides: 2.000. Boldfaced peptides were chosen as predicted epitopes.

**Table S2: Candidate HLA class I restricted HPV18 E6-specific CTL epitope predicted by algorithm as whole HPV18 E6 protein**

| HLA element | Epitope length | Epitope sequence | Position | Percentile Rank |
| --- | --- | --- | --- | --- |
| A*01:01 | 8 | N/A |  |  |
|  | 9 | FEDPTRRPY | aa4-12 | 1.183 |
|  |  | LTEVFEFAF | aa41-49 | 1.016 |
|  |  | QDIEITCVY | aa26-34 | 1.183 |
|  | 10 | LQDIEITCVY | aa25-34 | 0.421 |
|  |  | YSDSVYGDTL | aa81-90 | 0.965 |
|  |  | YSRIRELRHY | aa72-81 | 1.141 |
| A*02:01 | 8 | N/A |  |  |
|  | 9 | KLPDLCTEL | aa13-21 | 0.14 |
|  |  | SLQDIEITC | aa24-32 | 0.463 |
|  |  | FAFKDLFVV | aa47-55 | 0.557 |
|  |  | **GLYNLLIRC** | aa97-105 | 0.574 |
|  | 10 | **SLQDIEITCV** | aa24-33 | 0.114 |
|  |  | GLYNLLIRCL | aa97-106 | 0.587 |
|  |  | KLTNTGLYNL | aa92-101 | 0.985 |
| A*11:01 | 8 | N/A |  |  |
|  | 9 | **SVYGDTLEK** | aa84-92 | 0.002 |
|  |  | SIPHAACHK | aa59-67 | 0.62 |
|  |  | VVYRDSIPH | aa54-62 | 0.996 |
|  | 10 | DSVYGDTLEK | aa83-92 | 0.518 |
|  |  | SVYGDTLEKL | aa84-93 | 0.783 |
|  |  | LTEVFEFAFK | aa41-50 | 1.966 |
| A*24:02 | 8 | **N/A** |  |  |
|  | 9 | **VYGDTLEKL** | aa85-93 | 0.034 |
|  |  | VYCKTVLEL | aa33-41 | 0.049 |
|  |  | LYNLLIRCL | aa98-106 | 0.549 |
|  | 10 | VFEFAFKDLF | aa44-53 | 0.59 |
|  |  | SVYGDTLEKL | aa84-93 | 0.608 |
| B*07:02 | 8 | N/A |  | 4.19 |
|  | 9 | KPLNPAEKL | aa110-118 | 0.093 |
|  |  | NPAEKLRHL | aa113-121 | 0.098 |
|  |  | DPTRRPYKL | aa6-14 | 0.738 |
|  | 10 | LNPAEKLRHL | aa112-121 | 0.62 |
|  |  | QKPLNPAEKL | aa109-118 | 1.049 |
|  |  | RPYKLPDLCT | aa10-19 | 1.944 |
| B*40:02 | 8 | FEFAFKDL | aa45-52 | 0.341 |
|  |  | TEVFEFAF | aa42-49 | 1.224 |
|  | 9 | LEKLTNTGL | aa90-98 | 0.156 |
|  |  | FEFAFKDLF | aa45-53 | 0.201 |
|  |  | LELTEVFEF | aa39-47 | 0.315 |
|  | 10 | TLEKLTNTGL | aa89-98 | 1.078 |
|  |  | RELRHYSDSV | aa76-85 | 1.321 |
|  |  | TELNTSLQDI | aa19-28 | 1.576 |

Rank threshold for strong binding peptides: 0.500. Rank threshold for weak binding peptides: 2.000 Boldfaced peptides were chosen as predicted epitopes.
